# Structural changes of tubulin by interacting with Δ^9^-tetrahydrocannabinol: in-vitro and theoretical studies

**DOI:** 10.1186/s12868-025-00957-5

**Published:** 2025-07-30

**Authors:** Mina Mohammadkhani, Mostafa Jarah, Dariush Gholami, Gholamhossein Riazi, Hadi Rezazadeh

**Affiliations:** 1 Department of Biochemistry, Institute of Biochemistry and Biophysics (IBB), University of Tehran, Tehran, Iran; 2 Cinna Gen Medical Biotechnology Research Center, Alborz University of Medical Science, Karaj, Iran; 3 Department of Microbial Biotechnology, Faculty of Biotechnology, Amol University of Special Modern Technologies, Amol, Iran; 4 Faculty of Engineering Technology, Amol University of Special Modern Technologies, Amol, Iran

**Keywords:** Microtubule dynamicity, Δ^9^-tetrahydrocannabinol, Tubulin structure

## Abstract

**Background:**

There is growing evidence of the contribution of microtubule dynamics to dendritic spine changes, synaptic plasticity, axonal transportation, and cell polarity. Besides, one of the well-studied effects of *Cannabis* on human behavior is memory disability. As Δ^9^-tetrahydrocannabinol (Δ^9^-THC) is the most pivotal chemical of *Cannabis*, we investigated the effect of Δ^9^-THC on microtubule dynamicity and the structural study of tubulin (microtubule monomer).

**Results:**

Our results show that Δ^9^-THC changes microtubule dynamicity compared to the control group. The turbidity assay results demonstrated that Δ^9^-THC reduces microtubule polymerization in a concentration-dependent manner. Circular Dichroism spectroscopy also studied the structural changes of the purified tubulin, which revealed significant changes in the secondary structure of the tubulin. Furthermore, Silico studies predicted one binding site for Δ^9^-THC on β-tubulin.

**Conclusions:**

We concluded that Δ^9^-THC could reduce the microtubule’s stability, which may conversely affect brain function by microtubule dynamic changes caused by secondary structural changes of tubulin and preventing tubulin-tubulin interaction.

**Supplementary Information:**

The online version contains supplementary material available at 10.1186/s12868-025-00957-5.

## Graphical Abstract

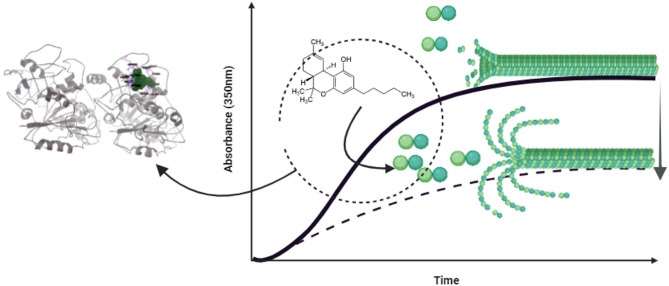

## Introduction

Microtubules are cytoskeletal filaments in eukaryotic cells, made up of α and β subunits [1], and are polarized polymeric tubes with dynamic ends [2]. Microtubules are essential for numerous aspects of the cell’s function. Cell morphogenesis, chromosome segregation, and intracellular vesicle transport are critical roles of microtubules [3, 4].

Furthermore, microtubules are critical structural and functional connectivity structures across the neural network and are involved in signal transportation along the axons [5, 6]. Dynamic microtubules are involved in dendritic spine changes and synaptic plasticity [7]. Microtubules’ motor proteins (dynein) are involved in the trafficking of intracellular subunits [8–10]. Different research groups have studied the structural changes that lead to the functional alteration of microtubules [11–13].

Δ^9^-Tetrahydrocannabinol (Δ^9^-THC) and cannabidiol (CBD), two major cannabinoids from Cannabis sativa, influence the cytoskeleton, particularly microtubules. Research on the direct interaction between the two ingredients and tubulin is limited. However, some studies have explored the effects of cannabinoids on the cytoskeleton, particularly microtubules composed of tubulin proteins. CBD has been observed to disrupt the organization of microtubules and microfilaments in PC12 cells, leading to a reduction in cytoskeletal integrity in a dose-dependent manner [14]. cannabinoid agonist affect acetylation of α-tubulin and gene expression in the prefrontal cortex of adult mice [15].

Δ^9^-THC is one of the most critical and psychoactive ingredients of *Cannabis.* It also impairs several aspects of cognitive functions, with the most robust effect on humans’ short-term, episodic, and working memory [16, 17].

The negative effect of Δ^9^-THC on synaptic plasticity is well-accepted [18, 19], and numerous studies have focused on this impairment’s signaling pathways, which are poorly understood [20–23]. Daniel Jimenez et al. reported that the activation of mouse astroglia type-1 cannabinoid receptors associated with mitochondrial membranes (mtCB1) disturbs glucose metabolism and lactate production in the brain. This leads to an alteration in neuronal functions [24]. Other results show that Δ^9^-THC-induced apoptosis in glioma cells may be independent of CB1 receptors and due to the THC-stimulated hydrolysis of sphingomyelin in glioma cells [25]. It has been shown that the long-term administration of Δ^9^-THC in laboratory animals impairs microtubule dynamicity [26].

According to previous studies on the direct role of microtubules in synaptic plasticity, cell structure, and the proven effects of Δ^9^-THC on the cell function, it can be presumed that Δ^9^-THC could exert its effect through changes in microtubule dynamicity along with other CB1 receptor-related mechanisms signaling pathway. To study this assumption, following our previous study of the systemic administration of Δ^9^-THC to rats [26], we examined the interaction between Δ^9^-THC and microtubule protein kinetically using a set of spectrophotometric techniques. Our results indicated that Δ^9^-THC has a binding site on tubulin and changes the dynamic behavior of the protein by inducing structural changes in the tubulin dimers.

## Materials and methods

### Chemicals

Δ^9^-THC (Cat# T4764) in methanol, prepared in a vehicle containing 4% DMSO and 96% saline (sterile saline 9%). Guanosine 5′-triphosphate (GTP, Cat# G0635), Piperazine-N, N′-bis (2-ethane sulfonic acid) (PIPES, Cat# P6757), EGTA **(**Cat# E3889), MgSO_4_ (Cat# E3889), Dimethyl sulfoxide (DMSO, Cat# D2650**)**, and other chemicals were purchased from Merck.

### Animals

Twenty-five adult Wistar rats, weighing 200–250 g, were purchased from the Institute of Biochemistry and Biophysics, University of Tehran, Tehran, Iran. The animals were kept under 12 h light/dark periods with access to standard food and water at the Neuroscience Research Center at the Institute of Biochemistry and Biophysics, University of Tehran, Tehran, Iran. The animals were anesthetized with an intraperitoneal injection of ketamine/xylazine (80 mg/kg and 10 mg/kg, respectively) as previously described [27]. The brain was quickly removed, and all brain tissue was fixed in a -80 °C for the next experiments, and the remaining carcasses were disposed of at the IBB animal care center.

### Rat brain tubulin extraction and purification

Tubulin was purified from the rat brain in two cycles of polymerization and depolymerization [28, 29]. For further purification, we applied cellulose phosphate column chromatography to remove MAPs for structural studies of the purified tubulins. The purity of the obtained fractions was determined by Coomassie blue staining of 10% sodium dodecyl sulfate-polyacrylamide gel electrophoresis (SDS-PAGE).

### Protein kinetics via turbidity measurement

The tubulin assembly assay was performed at 37 °C in the presence of a final concentration of 1 mM GTP solution in double-distilled water with a UV/visible spectrophotometer (at 350 nm wavelength), which was an adjustable temperature device [30]. To examine the effect of Δ^9^-THC on microtubule and tubulin assembly, the protein was pre-incubated with different concentrations of Δ^9^-THC for 2 min, and GTP was added to the solution to start the assay.

### Kinetic parameters of the tubulin polymerization

We followed the polymerization assay at 350 nm, as mentioned above. Information about the nucleation, lag phase, and elongation obtained from the polymerization curve [31] was assessed. The nucleation phase was studied with two different parameters, including (i) the tenth time or “t_1/10_” which refers to the necessary time to produce 1/10 of the final amount of the polymer, and (ii) parameter p, known as the number of successive steps in the nucleation process. The parameter p was used for estimating the nucleus size, which can be characterized by plotting log A_(t)_/A_max_ against log(t) where A_(t)_ is the absorbance at a given time and calculating the slope of the plot [32].

We also studied the elongation phase by plotting (1- A_t_ /A_max_) against the time, which provides a first-order rate constant of elongation (k_obs_). To analyze the tubulin assembly, we incubated the tubulin purified by cellulose phosphate column chromatography. Purified tubulin (30 µM) was incubated with different concentrations of Δ^9^-THC (0–1 µM) to test the direct interaction of Δ^9^-THC with tubulin in the absence of MAPs.

### Intrinsic fluorescence spectroscopy

The tendency of Δ^9^-THC to tubulins was determined by the intrinsic fluorescence of tubulins, which was detected in the presence of different concentrations (0–1 µM) of Δ^9^- THC. The emission of tubulins was detected at 295 nm excitation in the range of 300–500 nm [33]. The abovementioned experiments were carried out using the Cary Eclipse fluorescence spectrophotometer (Varian, Australia).

### Circular dichroism spectroscopy (CD)

The CD spectroscopy assay was performed by a model 215 circular Dichroism (Aviv Biomedical, USA). In this procedure, the tubulin dimers were pre-incubated with different concentrations of Δ^9^-THC (0–1 µM). The far UV CD spectra were recorded from 190 to 260 nm using a 1 mm path length quartz cuvette. Moreover, the secondary structural changes in tubulin were analyzed by the CDNN program.

### Molecular Docking studies

Molecular docking was performed using AutoDock tools version 1.5.6 with standard parameters. The crystal structure of tubulin was obtained from the Brookhaven Protein Data Bank (PDB entry 1JFF), and the ligand-free structure was used as the initial protein structure [34]. The ligand structure was obtained from Pub Chem (Pub Chem CID 16078). The grid box was defined (100_ 86 _74 Å) for a general pre-calculation of the interaction of Δ^9^-THC over the whole macromolecule, grid maps with a spacing of 0.375 Å in each direction, and the center of the grid was set to 19.17,0 and 0 Å. After recognizing the approximate binding site of Δ^9^-THC on tubulin, flexible docking of Δ^9^-THC over the β-tubulin binding site was performed. The grid box was defined as 40_40_40 Å, and the center of the grid was set to x = 3.641, y = -16.032, and z = 19.64. Images were created using Python Molecule Viewer (PMV), PyMol molecular viewer v.1.1, and the program Lig Plot v.1.0, which generates schematic 2-D representations of protein-ligand complexes from the PDB file input [35].

### Statistical analysis

The analysis of the data obtained was performed using SPSS software version 16. One-way analysis of variance (ANOVA) was performed, and Tukey post hoc analysis was performed to compare the means of the experimental groups. All results are expressed as mean ± SEM, and differences were considered significant at *P* < 0.05, *P* < 0.01, and *P* < 0.001.

## Results

### Rat brain tubulin extraction and purification

**Fig. 1:**
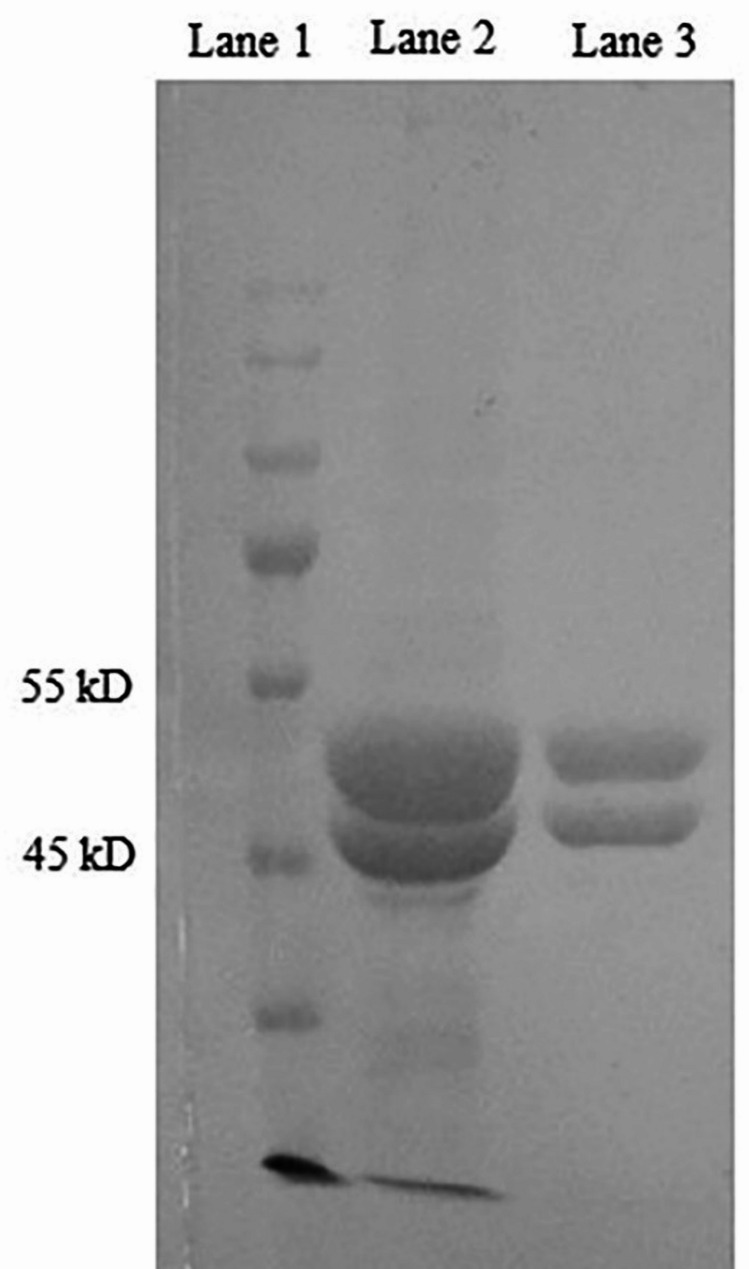
Coomassie brilliant blue-stained SDS-PAGE of the extracted and purified tubulin. Protein ladder (lane 1), tubulin dimers prepared from one cycle of polymerization and depolymerization of brain extract (lane 2), and purified tubulin (α-tubulin and β-tubulin) by phosphocellulose column chromatography (lane 3). The cropped ladder is used to improve the figure clarity

Tubulin dimers were obtained from two cycles of assembly and disassembly from the animals’ brain extracts, followed by a purification step with a phosphocellulose column (Fig. 1). MAP-free tubulin was successfully obtained, and a high level of purity was observed by sodium dodecyl sulfate-polyacrylamide gel (SDS-PAGE) stained by Coomassie Brilliant blue. As shown in Fig. 1, α-tubulin and β-tubulin are two separate narrow lines in lane 2.

### Δ^9^-THC significantly decreased tubulin polymerization with a significant effect on nucleation and elongation

**Fig. 2:**
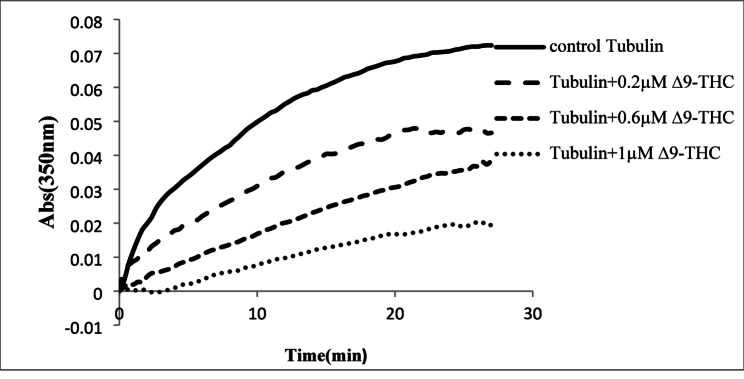
Increasing the concentrations of Δ^9^-THC significantly decreased the tubulin polymerization rate and maximum turbidity at the steady state

Turbidity measurements were conducted at 350 nm to characterize the effects of Δ^9^-THC on tubulin dynamicity (Fig. 2).

**Fig. 3:**
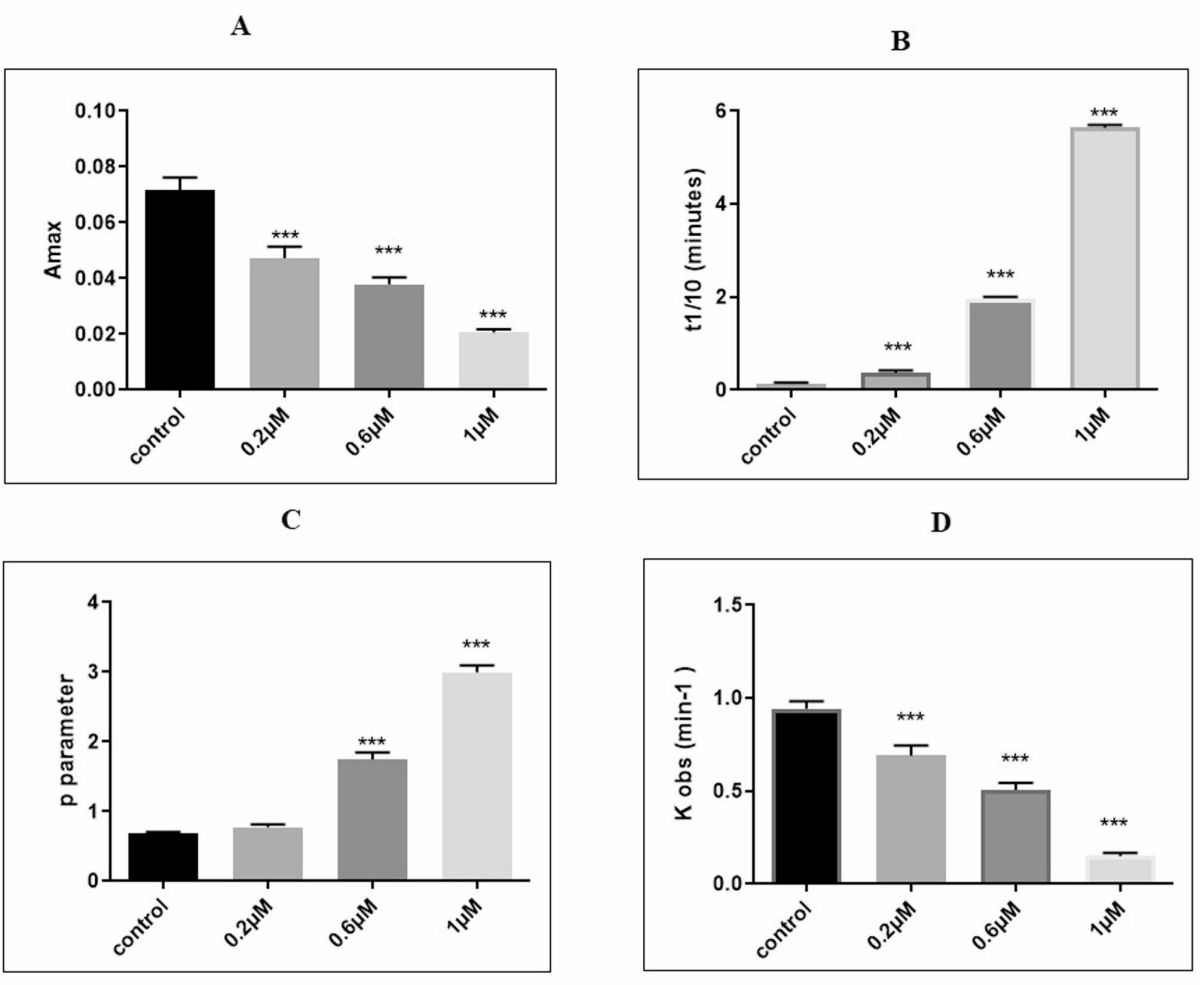
Kinetic parameters change significantly in purified tubulin interacting with different concentrations of Δ^9^-THC. **(A)** The maximum absorbance decreased with increasing concentrations of Δ^9^-THC. **(B)** The parameter t_1/10_ increased with higher concentrations of Δ^9^-THC, with the highest t_1/10_ observed at a concentration of 1 µM. Significant differences in t1/10 were observed between 0.6 µM and 0.2 µM concentrations (*p* < 0.001). **(C)** Δ^9^-THC significantly increased parameter p in a dose-dependent manner. The highest parameter p was observed significantly at a concentration of 1 µM (*p* < 0.001). **(D)** The observed rate constant (k_obs_) for purified tubulin was significantly reduced in Δ^9^-THC-treated tubulin compared with the control (*p* < 0.001). The lowest k_obs_ value was observed at a concentration of 1 µM (*p* < 0.05). The values represent the mean ± SEM. ***p < 0.001

The kinetic parameters calculated from the polymerization curve are presented in Table 1. The maximum absorbance (A_max_( at the steady state decreased significantly in a dose-dependent manner in the drug-treated samples as compared to the control (df = 11, F (3,8) = 119.1, *p* < 0.001, Fig. 3A). Δ^9^-THC significantly increased the tenth time of polymerization in all drug-treated tubulins (df = 11, F (3,8) = 114.56, *p* < 0.01 in 0.2 µM and *p* < 0.001 in 0.6, 1 µM Δ^9^-THC samples, Fig. 3B). Parameter p increased significantly in 0.6 and 1 Δ^9^-THC (df = 11, F (3, 8) = 61.3, *p* < 0.001, Fig. 3C), while k_obs_ decreased in a dose-dependent manner in Δ^9^-THC samples (df = 11, F (3, 8) = 207, *p* < 0.001, Fig. 3D).

**Table 1:** The polymerization and purification of tubulin depict the influence of different concentrations of Δ^9^-THC (0.2 ΜM, 0.6 ΜM, and 1 µM) on four relevant parameters: maximum absorbance (Amax), half-life (t1/10), parameter P, and the observed rate constant (k_obs_). It is noteworthy that Amax is reduced by increasing concentrations of Δ^9^-THC, whereas both t_1/10_ and parameter P exhibit dose-dependent enhancement. In contrast, k_obs_ fell off steeply with increasing Δ^9^-THC concentrations, reflecting a concentration-dependent Inhibition of the observed rate constant. This table gives a quantitative summary of the dose-response relation of Δ^9^-THC with the parameters measured, supplying information about its biochemical and kinetic action. *P < 0.05, **p < 0.01, ***p < 0.001; values represent the mean ± SEM

Δ^9^-THC concentration	A_max_	t_1/10_ (minutes)	parameter *p*	k_obs_
**Control**	0.071 ± 0.004	0.147 ± 0.016	0.68 ± 0.03	0.94 ± 0.04
**0.2 µM Δ** ^**9**^ **-THC**	0.048 ± 0.004**	0.37 ± 0.052*	0.77 ± 0.04	0.69 ± 0.055**
**0.6 µM Δ** ^**9**^ **-THC**	0.037 ± 0.002**	1.97 ± 0.03**	1.74 ± 0.100***	0.506 ± 0.037**
**1 µM Δ** ^**9**^ **-THC**	0.020 ± 0.001***	5.65 ± 0.529**	2.99 ± 0.11***	0.15 ± 0.016**

### Δ^9^-THC interacts with the tubulin dimer through one binding site

**Fig. 4:**
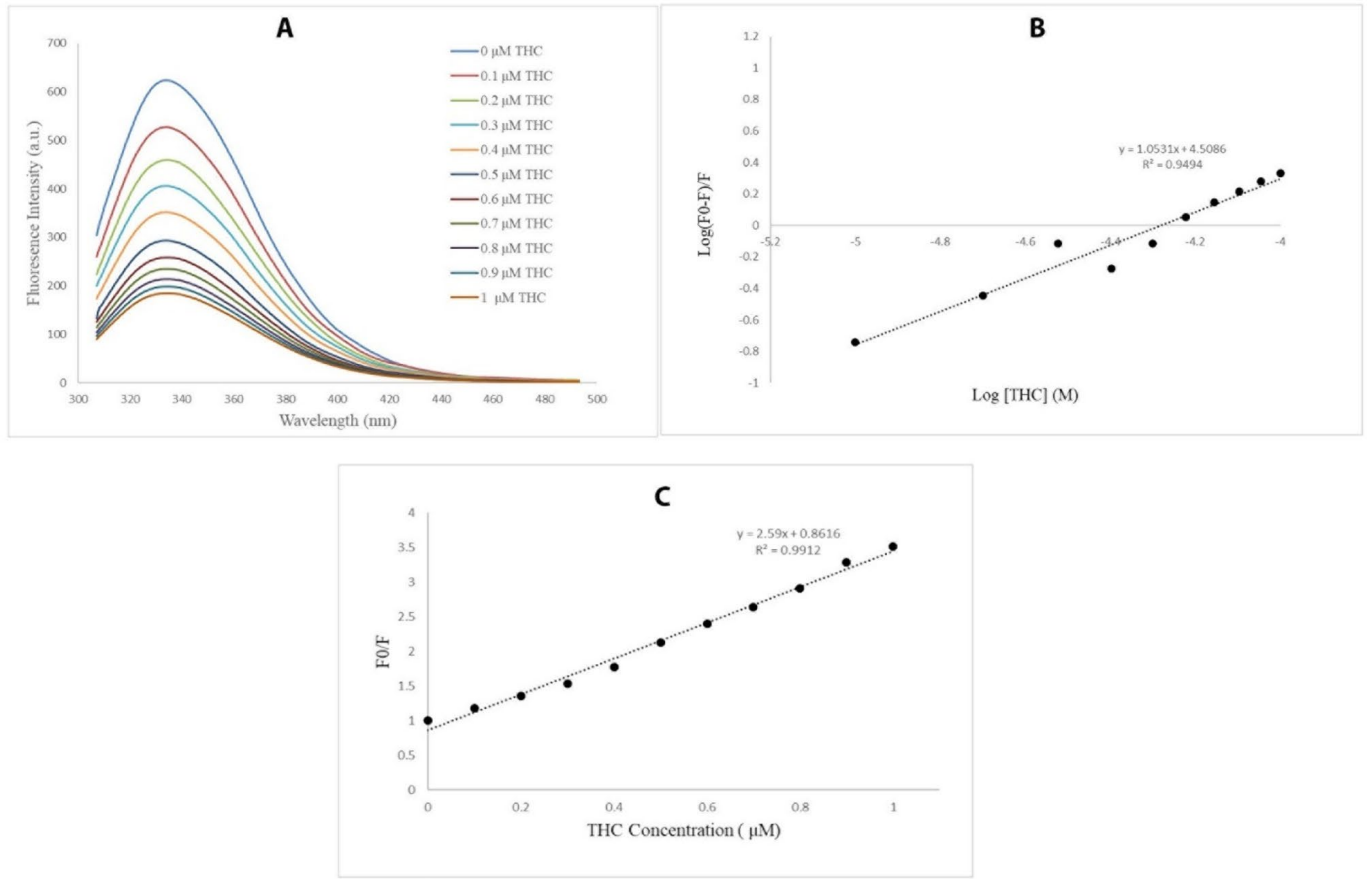
Intrinsic fluorescence binding of Δ^9^-THC to tubulin provides insights into the interaction mechanism between Δ^9^-THC and tubulin dimers. **(A)** Fluorescence quenching spectra of tubulins in the presence of various concentrations of Δ^9^-THC. The data obtained from the fluorescence intensity show that Δ^9^-THC interacted with tubulin dimers using a quenching mechanism, suggesting a direct binding interaction. **(B)** The numbers of binding sites (*n* = 1) of tubulin were obtained from the graph slope, and log *Ka* was derived from the plot of log [*F*0- *F /F*] against log [THC] (Ka = 3.16 × 10^4^). This confirms a specific and measurable binding affinity between Δ^9^-THC and tubulin. **(C)** Plotting F0/F against [THC] suggests a static interaction between tubulin and Δ^9^-THC with a quenching constant of 2.59 × 10^14^ L.s.mol^− 1^, further supporting the presence of a stable complex formation

Our results from the fluorescence titration indicated that Δ^9^-THC has a quenching effect on tubulin. The fluorescence emission intensity of tubulin decreased rapidly in the presence of increasing concentrations of Δ^9^-THC, an interaction between tubulin and Δ^9^-THC as shown in Fig. 4A. The number of binding sites and the binding constant were calculated by plotting log [ (F0–F)/F] against log [Δ^9^-THC] (Eq. 1), where F0 and F are the fluorescence intensities before and after the addition of the ligand, K_a_ is the binding constant, and [Q] is the concentration of the quencher.

This plot demonstrated that one binding site exists for Δ^9^-THC on the tubulin dimer and the binding constant is equal to 3.16 × 10^4^ L. mol^− 1^, indicating the tubulin’s high affinity for Δ^9^-THC as shown in Fig. 4B.

1 $$\:Log\left(\frac{{F}_{0}-F}{F}\:\right)=Log{K}_{a}+nLog\left[Q\right]$$

The Stern-Volmer equation (Eq. 2) was used to define the quenching constant. Kq, Ksv, and τ_0_ are the biomolecular quenching rate constants, the Stern–Volmer quenching constant, and the average lifetime of the biomolecule without the quencher (τ0 = 10^− 8^ s), respectively.

This plot indicated that the quenching constant was equal to 2.59 × 10^14^ L. s. mol^− 1^, suggesting a static interaction between Δ^9^-THC and tubulin (Fig. 4C). The fluorescence quenching mechanism is dynamic or static [36]. According to our data, the plot of Stern − Volmer is linear and the obtained K_q_ (2.59 × 10^14^ M^− 1^ S^− 1^) is larger than 2 × 10^10^ M^− 1^ S^− 1^ verifying that the main fluorescence quenching mechanism is likely static (Table 2).

2 $$\:\frac{{F}_{0}}{F}=1+{K}_{sv}\left[Q\right]=1+{K}_{q}{\tau}_{0}$$

**Table 2:** Biophysical parameters of Δ^9^-THC interaction with tubulin

K_a_ (M^− 1^)	K_sv_(M^− 1^)	K_q_ (M^− 1^ S^− 1^)	*n*
3.16 × 10^4^	2.59 × 10^6^	2.59 × 10^14^	1.053

### CD spectroscopy demonstrated a notably decreased α-helix in proportion to other secondary structures

**Fig. 5:**
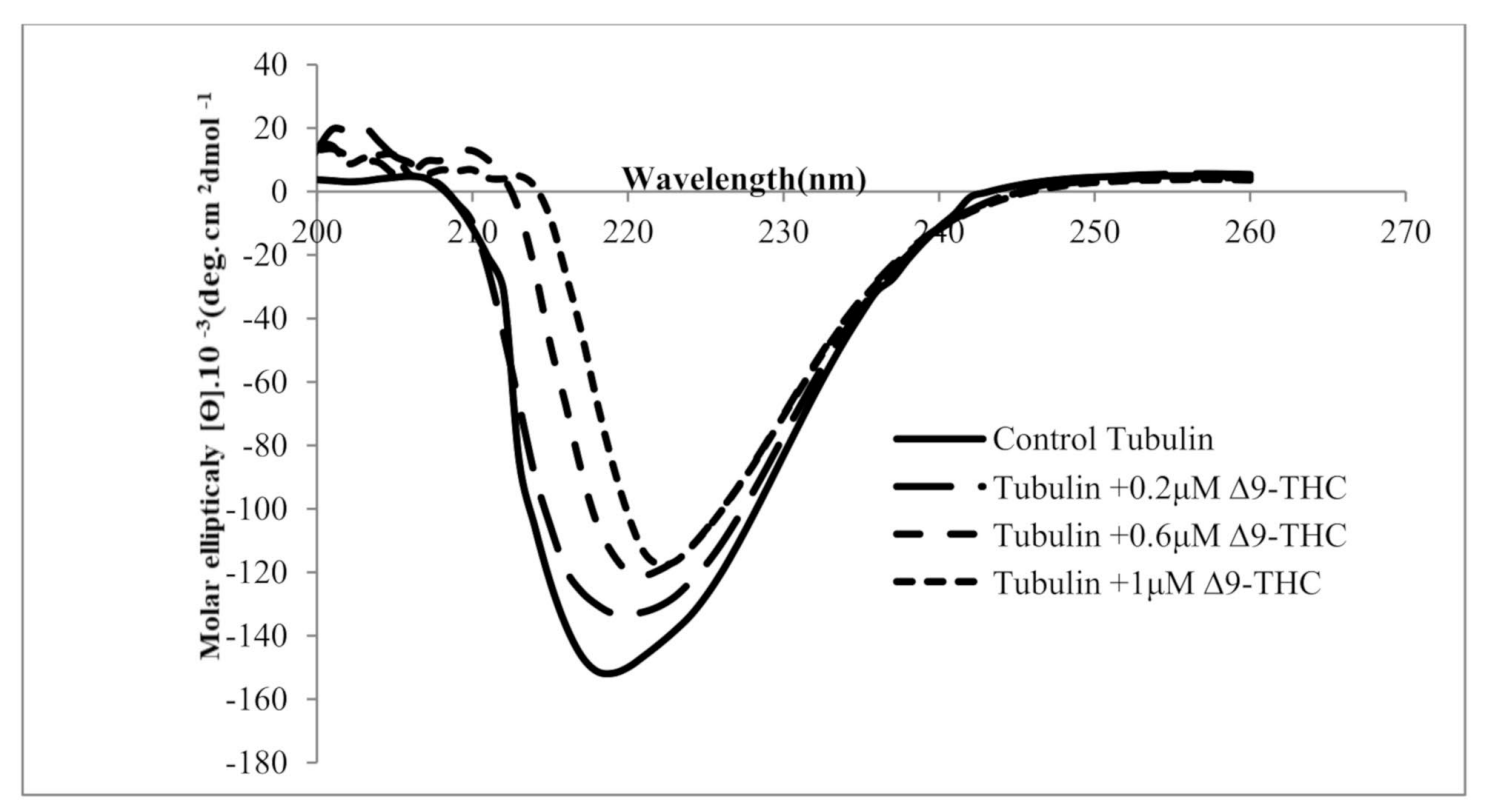
Effect of Δ^9^-THC on the secondary structure of tubulin. The secondary structural change of the tubulin dimers in the presence of various concentrations of Δ^9^-THC shows a reduction in the negative ellipticity at 220 nm

The CD data showed alterations in the secondary structure of tubulin (Fig. 5; Table 3). Decreasing intensity and broadening of both the positive and the negative CD bands led to a shift in the x-axis toward higher wavelengths and exhibited a slight red shift (Fig. 5).

The increase in β-sheet structure and decrease in α-helix coils were significantly observed in the drug-treated groups compared with the control sample (Table 3). α-helix secondary structure decreased significantly in Δ^9^-THC treated groups compared with the control (df = 11, F (3,8) = 58.9, *p* < 0.001). On the contrary, β-turn (df = 11, F (3,8) = 11.48, *p* = 0.0029), β-antiparallel (df = 11, F (3,8) = 75.12, *p* < 0.001), β-parallel (df = 11, F (3,8) = 174.1, *p* < 0.001), and random coil (df = 11, F (3,8) = 39.11, *p* < 0.001) increased significantly compared with the control group (Table 3).

**Table 3:** Alterations of the tubulin secondary structures in the presence of different concentrations of Δ^9^-THC. The decrease in α-helix structure was significantly more in the drug-treated groups than in the control group. The increase in β-turn, β-antiparallel, β-parallel, and the random coil was significantly observed in the drug-treated groups compared with the control group. *p < 0.05, **p < 0.01, ***p < 0.001; Values represent the mean ± SEM

Δ^9^-THC concentration	α-helix (%)	β-turn (%)	β-antiparallel (%)	β-parallel (%)	Random coil (%)
**Control**	52.6 ± 1.447	12.27 ± 0.688	3.93 ± 0.497	5.2 ± 0.251	21.73 ± 0.933
**0.2µM**	42.75 ± 1.824*	14.77 ± 0.95	6.1 ± 0.264**	6.76 ± 0.145**	28.53 ± 0.78**
**0.6µM**	30.47 ± 0.913***	15.20 ± 1.035**	8.13 ± 0.185***	8.76 ± 0.12***	31.97 ± 1.126**
**1µM**	24.02 ± 2.170***	19.07 ± 0.548**	10.93 ± 0.348***	10.67 ± 0.176***	36.73 ± 1.146**

### Docking studies

**Fig. 6:**
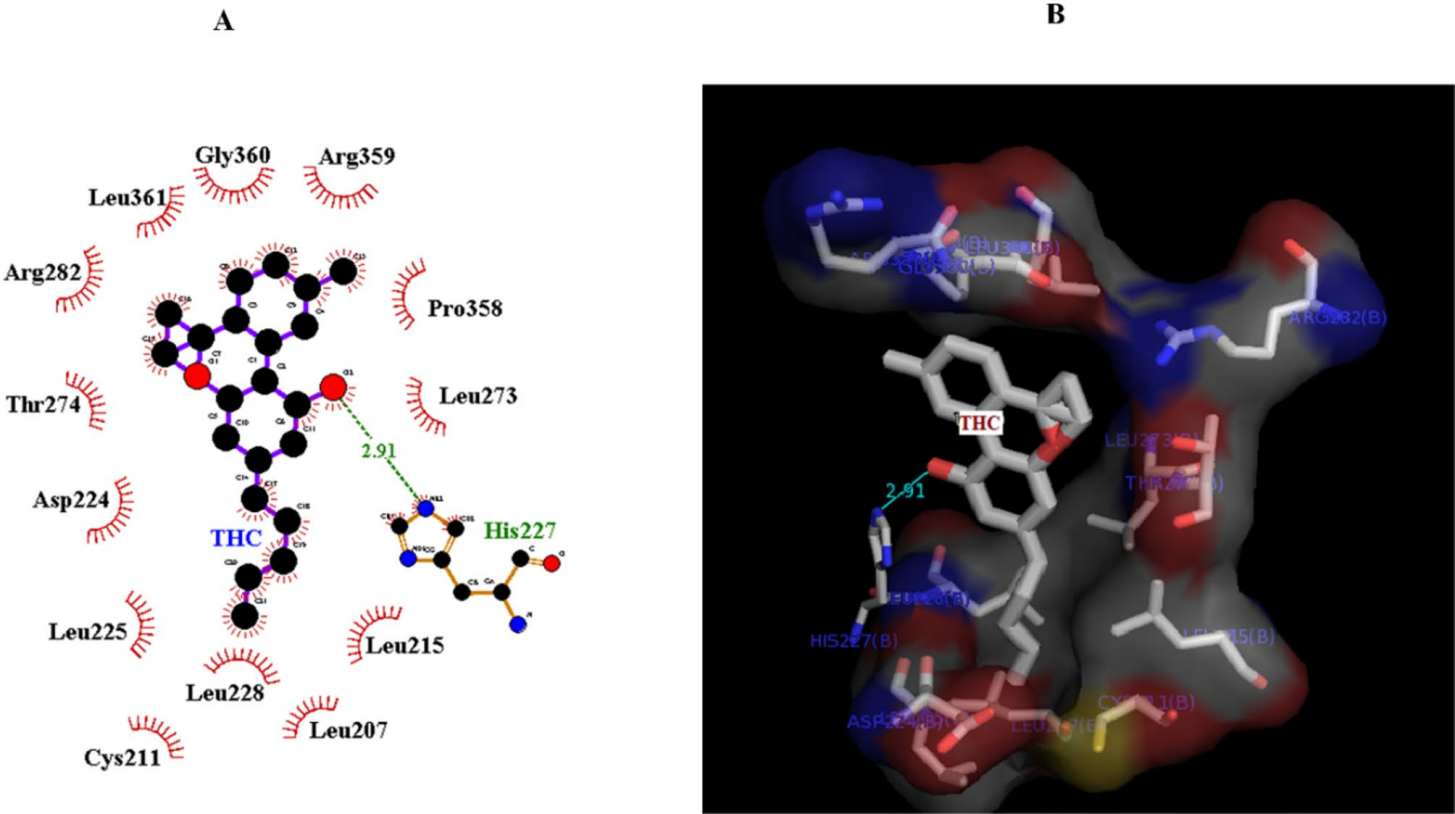
View of the tubulin-THC interaction. **(A)** The diagram plotted by the Lig plot shows the interaction between Δ^9^-THC and tubulin. Hydrophobic interactions play a significant role; one hydrogen bond (His227, with THC) is shown in green in the diagram. **(B)** The image was obtained using PyMol V1.1

**Fig. 7:**
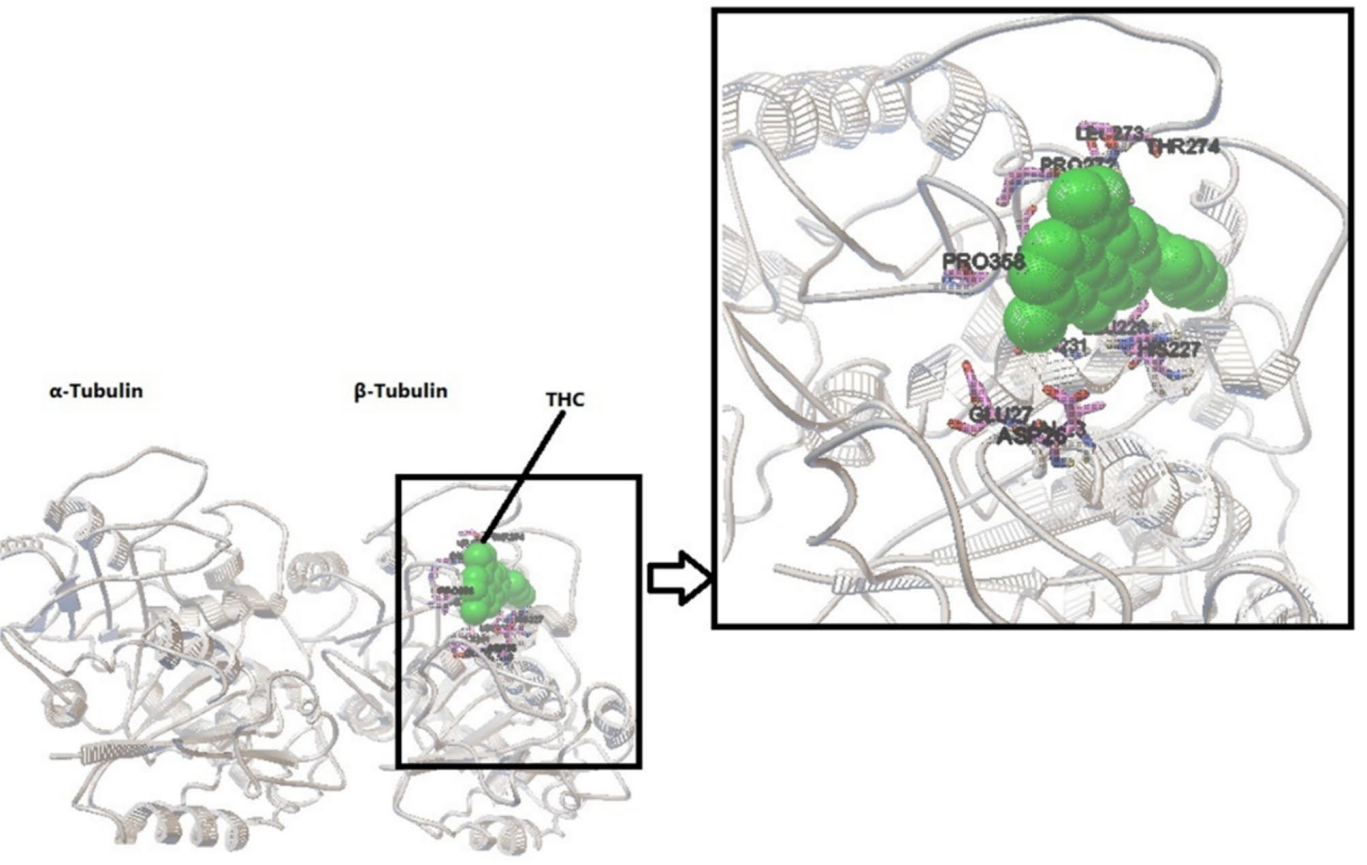
Stereo image presentation of the binding site of tubulin with Δ^9^-THC. Critical residues are shown in the zoom box, and THC is shown in green

Molecular docking provides insight into the binding of Δ^9^-THC to tubulin. The conformer with the lowest energy in the cluster, with 20 members of 400 conformers, was selected. According to AutoDock calculations, the binding energy of this structure was − 7 kcal/mol. A hydrogen bond between the side chain of His 227 and the OH group of the cyclohexane ring of Δ^9^-THC is orienting the ligand molecule to occupy the binding site. The results show that van der Waals interactions, hydrogen bonds, and hydrophobic interactions play a significant role in the binding of Δ^9^-THC to a hydrophobic pocket in β-tubulin. Based on the modeling studies, the dominant interaction is hydrophobic. As shown in Figs. 6A and B, the hydrophobic site of β-tubulin interacts with Δ^9^-THC via Arg359, Pro358, leu228, cys211, leu215, leu225, leu273, Thr 274, Arg 282, leu 361, leu207, and Gly360. According to the first docking over the entire tubulin, there is no binding site on α-tubulin, and all the full binding sites are on β-tubulin (Fig. 7). Δ^9^-THC mainly binds close to the GTP binding site on β-tubulin.

## Discussion

In this study, we proposed that Δ^9^-THC induces significant changes in the microtubule dynamicity resulting from the structural changes in the tubulin dimes.

Dynamic microtubules are involved in synaptic plasticity in the brain [7, 37]. Therefore, microtubule dysfunction may affect synaptic plasticity and spatial memory. Uv-visible spectroscopy represented that Δ^9^-THC exerts its effect through changes in microtubule dynamicity as well as, and microtubule dysfunction may affect synaptic plasticity as one, among others, possible mechanisms.

Studying the kinetics of the polymerization assay provided interesting data, increasing the tenth times (t_1/10_) demonstrates a more extended lag phase in Δ^9^-THC incubated samples compared to the control group. This suggests that the necessary time for tubulin dimer assembly in nucleation increased by Δ^9^-THC. In addition, the p parameter increased by increasing the Δ^9^-THC concentration, implying that Δ^9^-THC disturbs the lag phase and nucleation, while the k_obs_ value decreased strongly in a dose-dependent manner, showing that increasing Δ^9^-THC concentration has a critical role in reducing the elongation process of polymerization. Therefore, Δ^9^-THC suppresses elongation. It seems that Δ^9^-THC decelerates the nucleation and elongation process and reduces the final amount of the protein polymer.

The fluorescence intensity of the tubulin dimers decreased in the presence of Δ^9^-THC. As the Δ^9^-THC did not have any intrinsic fluorescence intensity, the fluorescence quenching seems to be the interactions between Δ^9^-THC and tubulin dimers, which is confirmed by previous studies [26]. The fluorescent studies and Stern-Volmer plot in this experiment show that there is a single stable site of Δ^9^-THC- tubulin interaction. The reduction of the fluorescence intensity originated from one of the static quenching mechanisms. Furthermore, the number of tubulin-binding sites (n) was calculated to be one. Three significant classes of tubulin-binding drugs have been identified: I; Colchicine binding ligands; ii, the Vinca domain drugs; and iii; Texans and Epothilone family. Vinca domain inhibitors, next to the nucleotide-binding region, inhibit polymerization by preventing nucleic acid exchange [38, 39]. Our computer-based study demonstrated one binding site for Δ^9^-THC on tubulin, which is around the Vinca region and makes a hydrogen bond with His 227 and electrostatic and hydrophobic interactions with hydrophobic amino acids on β-tubulin and might inhibit the GDP-GTP exchange and prevent microtubule polymerization.

Δ^9^-THC is a hydrophobic structure that contains three cyclic structures: a phenol ring, a pyran ring, and a cyclohexane ring, causing rigidity in the tubulin’s structure and the alkyl moiety [40]. Adding Δ^9^-THC to the tubulin solution might induce the exposure of hydrophobic amino acids normally buried inside α- helix secondary structures.

Our CD spectroscopy results elucidated that the proportion of α-helix structures was reduced in Δ^9^-THC treated compared to the control. Additionally, the proportion of β-sheets and other structures was increased in Δ^9^-THC treated in comparison with the control. This result is notably similar to previous in vitro studies [26]. The addition of Δ^9^-THC to the protein solution induces the secondary structures of the protein to transit to β- sheet and random coil structures, aiming to expose more hydrophobic amino acids. Thus, we predict that Δ^9^-THC can bind to tubulin dimers through hydrophobic interactions.

## Conclusions

In conclusion, our study reveals that Δ^9^-THC significantly disrupts microtubule dynamics by altering the structural properties of tubulin dimers, which may impair brain function. Δ^9^-THC extends the lag phase of tubulin assembly, reduces polymerization, and interacts with tubulin near the Vinca domain. Additionally, Δ^9^-THC induces a shift from α-helix to β-sheet and random coil structures, exposing the hydrophobic amino acids. These findings highlight the molecular mechanisms through which Δ^9^-THC affects neuronal functions. While this study provides insights into the direct interaction of Δ^9^-THC with tubulin, in vitro binding assays may not fully replicate cellular conditions. Our future studies will focus on cell culture studies to validate these findings.

## Electronic supplementary material

Below is the link to the electronic supplementary material.

Supplementary Material 1

## Data Availability

The data are available from the corresponding author upon reasonable request.

## References

[1] Dent EW. Of microtubules and memory: implications for microtubule dynamics in dendrites and spines. Mol Biol Cell. 2017;28(1):1–8. 28035040 10.1091/mbc.E15-11-0769 PMC5221613

[2] Akhmanova A, Steinmetz MO. Microtubule minus-end regulation at a glance. J Cell Sci. 2019;132(11):jcs227850. 31175152 10.1242/jcs.227850

[3] Akhmanova A, Steinmetz MO. Control of microtubule organization and dynamics: two ends in the limelight. Nat Rev Mol Cell Biol. 2015;16(12):711–26. 26562752 10.1038/nrm4084

[4] Foster PJ, Fürthauer S, Shelley MJ, Needleman DJ. From cytoskeletal assemblies to living materials. Curr Opin Cell Biol. 2019;56:109–14. 30500745 10.1016/j.ceb.2018.10.010

[5] Verstraelen P, Detrez JR, Verschuuren M, Kuijlaars J, Nuydens R, Timmermans J-P, De Vos WH. Dysregulation of microtubule stability impairs morphofunctional connectivity in primary neuronal networks. Front Cell Neurosci. 2017;11:173. 28690500 10.3389/fncel.2017.00173 PMC5480095

[6] Maday S, Twelvetrees AE, Moughamian AJ, Holzbaur EL. Axonal transport: cargo-specific mechanisms of motility and regulation. Neuron. 2014;84(2):292–309. 25374356 10.1016/j.neuron.2014.10.019 PMC4269290

[7] Jaworski J, Kapitein LC, Gouveia SM, Dortland BR, Wulf PS, Grigoriev I, Camera P, Spangler SA, Di Stefano P, Demmers J. Dynamic microtubules regulate dendritic spine morphology and synaptic plasticity. Neuron. 2009;61(1):85–100. 19146815 10.1016/j.neuron.2008.11.013

[8] Collingridge GL, Isaac JT, Wang YTJN. Receptor trafficking and synaptic plasticity. 2004, 5(12):952. 10.1038/nrn1556 15550950

[9] Anggono V, Huganir RL. Regulation of AMPA receptor trafficking and synaptic plasticity. Curr Opin Neurobiol. 2012;22(3):461–9. 22217700 10.1016/j.conb.2011.12.006 PMC3392447

[10] Chater TE, Goda Y. The role of AMPA receptors in postsynaptic mechanisms of synaptic plasticity. Front Cell Neurosci. 2014;8:401. 25505875 10.3389/fncel.2014.00401 PMC4245900

[11] Yousefzadeh SA, Jarah M, Riazi GH. Tryptophan improves memory independent of its role as a serotonin precursor: potential involvement of microtubule proteins. J Mol Neurosci. 2020;70:559–67. 31897970 10.1007/s12031-019-01457-y

[12] Atarod D, Eskandari-Sedighi G, Pazhoohi F, Karimian SM, Khajeloo M, Riazi GH. Microtubule dynamicity is more important than stability in memory formation: an in vivo study. J Mol Neurosci. 2015;56:313–9. 25740015 10.1007/s12031-015-0535-4

[13] Mohammadkhani M, Gholami D, Riazi G. The effects of chronic morphine administration on Spatial memory and microtubule dynamicity in male mice’s brain. IBRO Neurosci Rep. 2024;16:300–8. 38390235 10.1016/j.ibneur.2024.02.002 PMC10881431

[14] Tahir SK, Trogadis JE, Stevens JK, Zimmerman AM. Cytoskeletal organization following cannabinoid treatment in undifferentiated and differentiated PC12 cells. Biochem Cell Biol. 1992;70(10–11):1159–73. 1297339 10.1139/o92-162

[15] Tomas-Roig J, Ramasamy S, Zbarsky D, Havemann-Reinecke U, Hoyer-Fender S. Psychosocial stress and cannabinoid drugs affect acetylation of α-tubulin (K40) and gene expression in the prefrontal cortex of adult mice. PLoS ONE. 2022;17(9):e0274352. 36129937 10.1371/journal.pone.0274352 PMC9491557

[16] Ranganathan M, D’Souza DC. The acute effects of cannabinoids on memory in humans: a review. Psychopharmacology. 2006;188(4):425–44. 17019571 10.1007/s00213-006-0508-y

[17] Sullivan JM. Cellular and molecular mechanisms underlying learning and memory impairments produced by cannabinoids. Learn Mem. 2000;7(3):132–9. 10837502 10.1101/lm.7.3.132

[18] Wise LE, Thorpe AJ, Lichtman AH. Hippocampal CB 1 receptors mediate the memory impairing effects of Δ 9-tetrahydrocannabinol. Neuropsychopharmacology. 2009;34(9):2072–80. 19322169 10.1038/npp.2009.31 PMC2822461

[19] Clarke JR, Rossato JI, Monteiro S, Bevilaqua LR, Izquierdo I, Cammarota M. Posttraining activation of CB1 cannabinoid receptors in the CA1 region of the dorsal hippocampus impairs object recognition long-term memory. Neurobiol Learn Mem. 2008;90(2):374–81. 18524639 10.1016/j.nlm.2008.04.009

[20] Puighermanal E, Marsicano G, Busquets-Garcia A, Lutz B, Maldonado R, Ozaita A. Cannabinoid modulation of hippocampal long-term memory is mediated by mTOR signaling. Nat Neurosci. 2009;12(9):1152–8. 19648913 10.1038/nn.2369

[21] Derkinderen P, Valjent E, Toutant M, Corvol J-C, Enslen H, Ledent C, Trzaskos J, Caboche J, Girault J-A. Regulation of extracellular signal-regulated kinase by cannabinoids in hippocampus. J Neurosci. 2003;23(6):2371–82. 12657697 10.1523/JNEUROSCI.23-06-02371.2003 PMC6742049

[22] Ozaita A, Puighermanal E, Maldonado R. Regulation of PI3K/Akt/GSK-3 pathway by cannabinoids in the brain. J Neurochem. 2007;102(4):1105–14. 17484726 10.1111/j.1471-4159.2007.04642.x

[23] Maroso M, Szabo GG, Kim HK, Alexander A, Bui AD, Lee S-H, Lutz B, Soltesz I. Cannabinoid control of learning and memory through HCN channels. Neuron. 2016;89(5):1059–73. 26898775 10.1016/j.neuron.2016.01.023 PMC4777634

[24] Jimenez-Blasco D, Busquets-Garcia A, Hebert-Chatelain E, Serrat R, Vicente-Gutierrez C, Ioannidou C, Gómez-Sotres P, Lopez-Fabuel I, Resch-Beusher M, Resel E, et al. Glucose metabolism links astroglial mitochondria to cannabinoid effects. Nature. 2020;583(7817):603–8. 32641832 10.1038/s41586-020-2470-y

[25] Sánchez C, Galve-Roperh I, Canova C, Brachet P, Guzmán M. Δ^9^-Tetrahydrocannabinol induces apoptosis in C6 glioma cells. FEBS Lett. 1998;436(1):6–10. 9771884 10.1016/s0014-5793(98)01085-0

[26] Gholami D, Noori AR, Mohammadkhani M, Emruzi Z, Riazi GH. The long-term effects of Δ^9^-tetrahydrocannabinol on microtubule dynamicity in rats. Arch Biochem Biophys. 2020;693:108574. 32898566 10.1016/j.abb.2020.108574

[27] Choi KH, Whisler K, Graham DL, Self DW. Antisense-induced reduction in nucleus accumbens Cyclic AMP response element binding protein attenuates cocaine reinforcement. Neuroscience. 2006;137(2):373–83. 16359811 10.1016/j.neuroscience.2005.10.049

[28] Miller HP, Wilson L. Preparation of microtubule protein and purified tubulin from bovine brain by cycles of assembly and disassembly and phosphocellulose chromatography. Methods in cell biology. Volume 95. edn.: Elsevier; 2010. pp. 2–15. 10.1016/S0091-679X(10)95001-2 20466126

[29] Gholami D, Riazi G, Fathi R, Sharafi M, Shahverdi A. Comparison of polymerization and structural behavior of microtubules in rat brain and sperm affected by the extremely low-frequency electromagnetic field. BMC Mol Cell Biology. 2019;20(1):41. 10.1186/s12860-019-0224-1 PMC6716927 31464580

[30] Shelanski ML, Gaskin F, Cantor CR. Microtubule assembly in the absence of added nucleotides. Proc Natl Acad Sci U S A. 1973;70(3):765–8. 4514990 10.1073/pnas.70.3.765 PMC433354

[31] Johnson KA, Borisy GG. Kinetic analysis of microtubule self-assembly in vitro. J Mol Biol. 1977;117(1):1–31. 599563 10.1016/0022-2836(77)90020-1

[32] Bonfils C, Bec N, Lacroix B, Harricane M-C, Larroque C. Kinetic analysis of tubulin assembly in the presence of the microtubule-associated protein TOGp. J Biol Chem. 2007;282(8):5570–81. 17178729 10.1074/jbc.M605641200 PMC2238798

[33] Chang G-G, Lee H-J. Monitoring protein conformational changes by quenching of intrinsic fluorescence. J Biochem Biophys Methods. 1984;9(4):351–5. 6491156 10.1016/0165-022x(84)90019-8

[34] Word JM, Lovell SC, Richardson JS, Richardson DC. Asparagine and glutamine: using hydrogen atom contacts in the choice of side-chain amide orientation1. J Mol Biol. 1999;285(4):1735–47. 9917408 10.1006/jmbi.1998.2401

[35] Wallace AC, Laskowski RA, Thornton JM. LIGPLOT: a program to generate schematic diagrams of protein-ligand interactions. Protein Eng. 1995;8(2):127–34. 7630882 10.1093/protein/8.2.127

[36] Ding J, Yuan L, Gao L, Chen J. Fluorescence quenching of a Rhodamine derivative: selectively sensing Cu2 + in acidic aqueous media. J Lumin. 2012;132(8):1987–93.

[37] Dent EW. Dynamic microtubules at the synapse. Curr Opin Neurobiol. 2020;63:9–14. 32062144 10.1016/j.conb.2020.01.003 PMC7423735

[38] Cormier A, Marchand M, Ravelli RB, Knossow M, Gigant, BJEr. Structural insight into the Inhibition of tubulin by vinca domain peptide ligands. EMBO Rep. 2008;9(11):1101–6. 18787557 10.1038/embor.2008.171 PMC2581847

[39] Mitra A, Sept D. Localization of the antimitotic peptide and depsipeptide binding site on beta-tubulin. Biochemistry. 2004;43(44):13955–62. 15518544 10.1021/bi0487387

[40] Borges RS, Batista J, Viana RB, Baetas AC, Orestes E, Andrade MA, Honório KM, Da Silva AB. Understanding the molecular aspects of tetrahydrocannabinol and Cannabidiol as antioxidants. Molecules. 2013;18(10):12663–74. 24129275 10.3390/molecules181012663 PMC6269679

