# Supplementary material for: Structural changes of tubulin by interacting with Δ9-tetrahydrocannabinol: in-vitro and theoretical studies
Source: BMC Neurosci. 2025 Jul 30;26:47. doi: 10.1186/s12868-025-00957-5 (PMC12312531; doi:10.1186/s12868-025-00957-5)

Structural changes of tubulin by interacting with Δ^9-tetrahydrocannabinol: *in-vitro* and theoretical studies

Mina Mohammadkhani[1], Mostafa Jarah[1, 2], Dariush Gholami[3*], Gholamhossein Riazi[1,] Hadi Rezazadeh[4]

*Correspondence: d.gholami@ausmt.ac.ir
[3] Faculty of Biotechnology, Amol University of Special Modern Technologies, Amol, Iran

Supplementary Figure 1. Full-length gel is presented in Supplementary Figure 1. Coomassie brilliant blue-stained SDS-PAGE of the extracted and purified tubulin. Protein ladder (lane 1), tubulin dimers prepared from one cycle of polymerization and depolymerization of brain extract (lane 2), and purified tubulin (α-tubulin and β-tubulin) by phosphocellulose column chromatography (lane 3). The cropped ladder is used to improve the figure clarity.

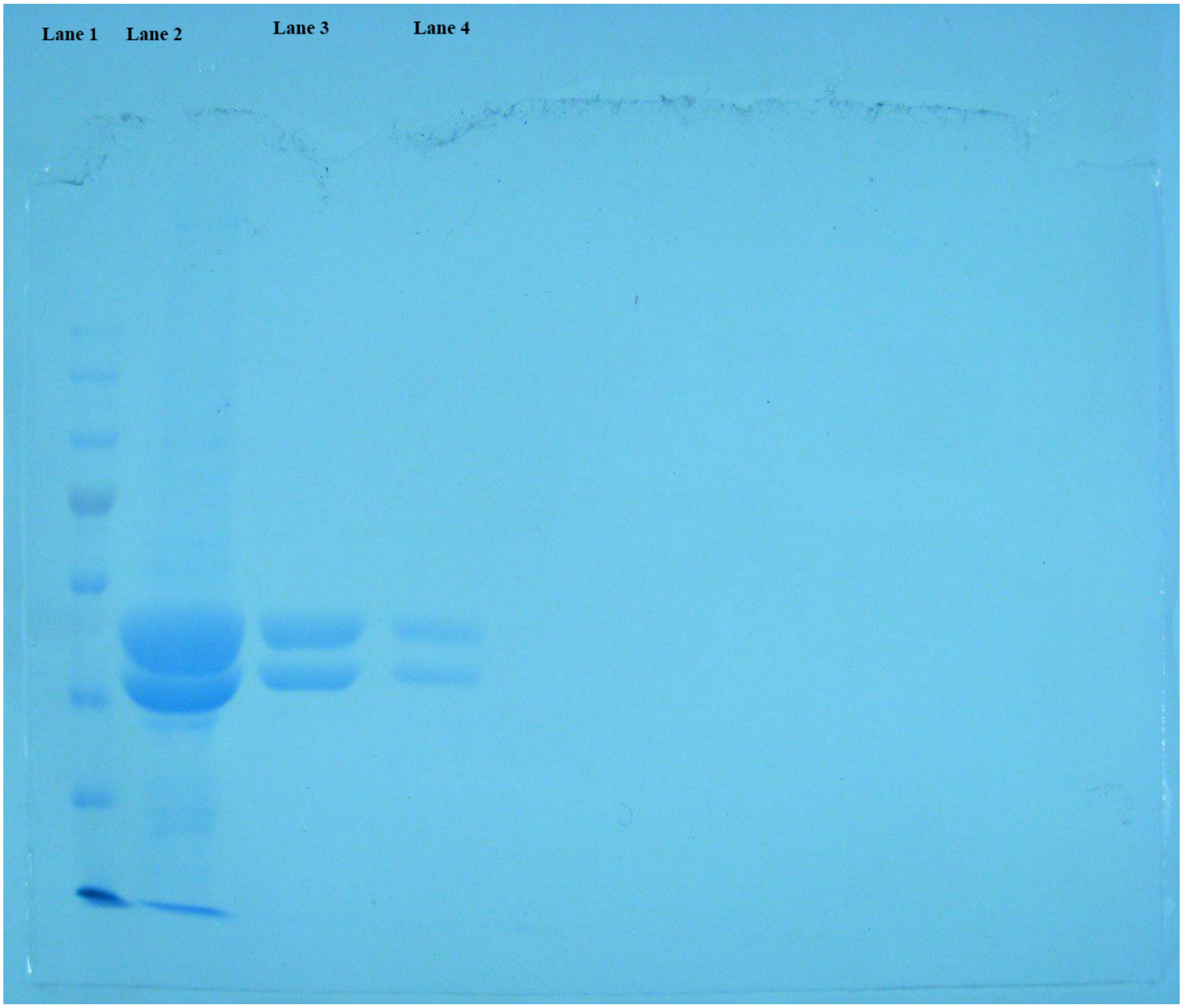

Supplement: Supplementary file 1 — Supplementary Material 1 [file 12868_2025_957_MOESM1_ESM.pdf]
